# KODY, an all-of-family response to co-occurring substance use and domestic violence: protocol for a quasi-experimental intervention trial

**DOI:** 10.1186/s12889-022-12529-x

**Published:** 2022-02-12

**Authors:** Margaret Kertesz, Cathy Humphreys, Larissa Fogden, Katreena Scott, Anne-Marie Laslett, Menka Tsantefski

**Affiliations:** 1 Department of Social Work, University of Melbourne, Victoria, 3010 Australia; 2 Faculty of Education, Western University, London, Ontario, N6G 1G7 Canada; 3 Centre for Alcohol Policy Research, La Trobe University, Melbourne, Victoria, 3086 Australia; 4 School of Health Sciences and Social Work, Griffith University, Gold Coast, Queensland, 4222 Australia

**Keywords:** Domestic violence, Father, Intervention, Quasi-experimental, Parenting, Child welfare, Perpetrator

## Abstract

**Background:**

The co-occurrence of domestic violence with alcohol and other drugs significantly increases the severity of abuse and violence experienced by family members. Longitudinal studies indicate that substance use is one of few predictors of men’s continued use of, or desistance from, violence. Recent developments in men’s behaviour change programs have focused on men’s attitudes and behaviour towards their children, and the exploration of interventions that address the needs of all family members. However, the research evidence is limited on the most effective elements of men’s behaviour change programs in promoting the safety and wellbeing of child and women victim survivors.

This study aims to build on the existing evidence by trialling the KODY program which addresses harmful substance use by men who also perpetrate domestic violence; the safety and wellbeing of women and children; the needs of children in their own right, as well as in relationship with their mothers; and the development of an ‘all-of-family’ service response. The evaluation of these innovations, and the ramifications for policy development to support less fragmented service system responses, provide the rationale for the study.

**Methods/Design:**

A quasi-experimental design will be used to assess the primary outcomes of improving the safety and wellbeing of mothers and children whose (ex)partners and fathers respectively participate in KODY (the trial program), when compared with ‘*Caring Dads* standard’ (the comparison group). Psychometric tests will be administered to fathers and mothers at baseline, post-program and at 3-month follow up. Data collection will occur over three years.

**Discussion:**

By building the evidence base about responses to co-occurring domestic violence and substance use, this study aims to develop knowledge about improving safety outcomes for women and children, and to better understand appropriate support for children in families living at the intersection of domestic violence and substance use. It is anticipated that study findings will point to the ramifications for policy development to support less fragmented service system responses.

**Trial registration:**

An application for registration with the Australian and New Zealand Clinical Trials Registry (https://www.anzctr.org.au/) was lodged on 20 December 2021 (Request number: 383206)—prospectively registered.

## Background

The co-occurrence of domestic violence (DV) with alcohol and other drugs (AOD) significantly increases the severity of abuse and violence experienced by family members [1]. Programs for men who use violence generally focus on addressing the violence and abuse directed at women (their partners or ex-partners), while children, who make up 50% of those affected, are rarely in focus [2].

A new wave of programs for men who use violence which focus on their fathering, attitudes and behaviour towards their children, has seen *Caring Dads* programs developed by Scott, Kelly, Crooks and Francis (2018) in Canada, and adapted and evaluated in Australia and internationally [3–7]. The recent evaluation of the *Caring Dads* program in Australia identified a range of issues which the proposed innovative program and evaluation seeks to address to improve health and wellbeing outcomes and the effectiveness of the program. These include a) the problems associated with the co-occurrence of DV with AOD issues; b) the requirement to develop safe ‘all-of-family’ responses, particularly for families who are not separating; c) the lack of attention to the support needs of children whose fathers are attending *Caring Dads*; and d) the requirement for evaluation measures for programs to address fathering, DV and AOD.

The quasi-experimental trial discussed in this paper was designed to pilot and evaluate an all-of-family response to the complex issues for children, women and men living with AOD and DV. The KODY program (Kids First (*Caring Dads*) and Odyssey House Victoria) integrates and enhances the work of two non-government organisations in Melbourne, Australia. Kids First Australia auspices a *Caring Dads* program. Odyssey House Victoria provides a range of alcohol and other drug treatment programs including services for parents affected by problematic substance use and their children, such as *Kids in Focus*, a family centred program that emphasises the safety and wellbeing of children in addition to providing parenting and family support.

Victorian domestic homicide reviews indicate that between 50–60% of murders occur in the context of the use of alcohol or other drugs [8] suggesting an association between the severity of DV and the use of AOD. In longitudinal studies, AOD use has emerged as one of few consistent predictors of men’s continued use of, or desistance from violence [9, 10]. Substance use is also associated with negative, hostile and aggressive parenting [11, 12]. The Australian *Caring Dads* evaluation for fathers who use violence found that a third of participants were accessing AOD services [6]. Some groups are over-represented in their need for these services. A significant proportion of the *Kids in Focus* clients at Odyssey House Victoria come from Aboriginal or culturally and linguistically diverse (CALD) backgrounds. In addition, a considerable number of men display co-occurring mental health issues [13]. Unemployed men who hold highly traditional notions of masculinity are also over-represented among users of harmful alcohol use and DV [10, 14, 15]. This body of research suggests the need to explore models of intervention that address both men’s substance use and their use of violence and coercive control [12].

There is widespread recognition that DV is predominantly a gendered issue of men’s violence towards women [2]. However, less attention has been given to children as victims of the destructive parenting behaviours of fathers who use domestic violence and coercive control [16]. The evidence is compelling and well established that children’s exposure has negative consequences for them [17]. These include adverse health impacts [18], the undermining of children’s emotional and psychological wellbeing [19], and problems connected with damaging behaviours. Similarly, the destructive impact for children living with parental harmful substance use is a significant driver of children into child protection and out of home care [20], particularly when there is co-occurrence with DV and carer mental ill-health [21, 22].

Despite the established importance of DV and AOD to child outcomes, comparatively little notice has been taken of attitudes and behaviours towards children in interventions for either of these problems. AOD programs, although well researched for their efficacy regarding reduced use of substances, have very seldom considered or included improved fathering and better child outcomes as a program or evaluation outcome. Recent overviews of research on men’s behaviour change (MBC) programs stress the need for robust evaluations of more targeted programs that respond to the diverse needs and characteristics of the men referred [23, 24]. Currently, it is unclear which elements of these programs work for which men, under what circumstances, and how they contribute to increased safety for women and children. There is evidence that some men are motivated by their desire to be a good father and that this can be a source of behaviour change [25]. Evaluations of the small number of programs for fathers who use violence show positive findings [3–7, 26].

A more recent, although still early, development in the area of MBC programming [27, 28] and other interventions in the DV area [29] has been the exploration of interventions for all family members. ‘All-of-family’ programs address the needs of each family member in their own right [26], going beyond the often-marginalised partner support component attached to MBC or *Caring Dads* programs and focusing on children as well as women victim survivors. The ‘all-of-family’ model is a DV-informed framework developed by the Safe and Together Institute to address the needs and responses to each family member living at the intersection of DV and AOD [30]. This approach is particularly appropriate when families are not in a position to separate [28, 31].

Through assessing the effectiveness of the innovative KODY program, this study aims to address many of these issues: harmful substance use by men who also perpetrate DV; the needs of children in their own right, as well as in relationship to their mothers through the Odyssey House Victoria *Kids in Focus* program; and the development of an ‘all-of-family’ service response. The evaluation of these innovations and the ramifications for policy development to support less fragmented service system responses provide the rationale for the proposed trial.

## Methods / Design

The primary aim of the study is to test the effectiveness of the KODY program in supporting safety for mothers and children with experiences of DV perpetrated by men with problematic substance use. It is hypothesised that the program will:

1) improve the safety and wellbeing of women whose (ex)partners participate in KODY *Caring Dads*;

2) improve the safety and wellbeing of children, whose fathers participate in KODY *Caring Dads*.

Secondary outcomes include fathers’ use of DV and of AOD (see Table 1).

**Table 1:** Primary and Secondary Outcomes and measures

Outcomes measured	Outcomes Measures completed by Fathers	Outcomes Measures completed by Mothers
**Primary Outcomes**		
** Safety and well-being of women**		Composite Abuse Scale
** Safety and well-being of children**	Emotional Dysregulation Scale	Emotional Dysregulation Scale
	Interpersonal Mindfulness in Parenting Scale	Brief Infant–Toddler Social and Emotional Assessment
		* Strengths and Difficulties Questionnaire – ages 3 +
**Secondary Outcomes**		
** Fathers’ use of DV**	Mirabal measures (adapted)	Mirabal measures (adapted)
	Anger Management Scale	Composite Abuse Scale
	Brief Irritability Test	
** Fathers’ substance use**	Alcohol Use Disorders Identification Test (AUDIT)	
	Drug Use Disorders Identification Test (DUDIT)	

^*^ Adolescents involved with KODY will also be invited to fill in the appropriate version of the Strengths and Difficulties Questionnaire

The effectiveness of the KODY program will be tested with a quasi-experimental design comparing outcomes between KODY (the trial program) and ‘*Caring Dads* standard’ [32] (the comparison group). Due to the small-scale and innovative nature of the trial program, it was deemed impracticable and unethical to randomly assign participants to trial and comparison groups. Psychometric tests will be administered to men and women participants at three timepoints—baseline (T1), post-*Caring Dads* program (T2) and three months after the end of the *Caring Dads* program (T3). Supplementary analyses of participation data obtained through file reviews will be conducted to examine the extent to which intervention effects vary according to ‘dose’ level of expanded service. In the context of this trial, ‘dose’ levels refer to the number of group work sessions attended by fathers, the degree of engagement by mothers with the Child and Family Wellbeing Practitioner and the *Kids in Focus* activities in which children, mothers and fathers participate.

The program and outcomes measures will first be tested for feasibility. To add context to the findings of the quasi-experimental study, a process evaluation will also be conducted, drawing on interviews with children, women and men, as well as program administrative data. Data collection will occur over three years.

### The intervention

The trial intervention (KODY) is a new model of service that integrates the work of both Kids First and Odyssey House Victoria to address the complex issues for children, women and men living with AOD and DV. The ‘*Caring Dads* standard’ program (the comparison group) consists of the following components.

**Group work**—17 sessions, manualised content, focus on engaging men to examine their fathering, increasing child centred fathering, including respect and support of children’s mothers, and changing patterns of harmful fathering.

**Case Coordination**—case coordination between group intervention and mother contact practitioners.

**Services for mothers and children**—mothers of children with whom fathers have contact are provided support and safety planning through a Child and Family Wellbeing Practitioner.

KODY (the trial program) comprises the ‘*Caring Dads* standard’ program plus the following changes and additions.

**Group work**—‘*Caring Dads* standard’ program with added curriculum materials and specialist AOD facilitation of group sessions.

**Case Coordination**—Collaborative case coordination for fathers by KODY DV and AOD practitioners. Additionally, *Kids in Focus* offers all-of-family case management, which will be integrated with *Caring Dads* case coordination.

**Services for mothers and children**—In addition to support from the Child and Family Wellbeing Practitioner, linked intervention with all family members (children, mothers and fathers) through *Kids in Focus*, including parenting education and support and recreational and therapeutic groups for children and their families.

The intervention group will be implemented by a co-facilitation team with expertise in working with fathers to address DV and AOD. The comparison group will be implemented by professionals experienced in DV and the delivery of *Caring Dads*. Adherence to intervention protocols will be monitored through regular meetings with program staff, recording of participation across time and program components, and observation of selected KODY *Caring Dads* group sessions.

### Eligibility criteria

The target group for study is fathers who use DV and AOD. Fathers and their (ex)partners eligible to participate in either the trial or the comparison programs will be eligible for the study. In both groups, eligible fathers must have contact with at least one child, be able to understand and speak English, and have capacity to maintain participation and attend group work regularly, bearing in mind their substance use status. Mothers participating in trial and comparison programs are also eligible for the study.

The trial program (KODY) will constitute fathers referred for their problematic AOD use and identified by AOD case managers as using DV, or at risk of using DV. KODY fathers must be receiving support through Odyssey House Victoria clinicians. These men, their children with whom they have contact, and the children’s mothers, will be offered support through *Kids in Focus* as part of the intervention group. The comparison group will comprise fathers who are referred (or self-refer) to a ‘*Caring Dads* standard’ program. These fathers will have been identified as using DV or being at risk of using DV.

### Recruitment and sampling

Recruitment into KODY and into ‘*Caring Dads* standard’ program will occur through referrals from a range of services, including other programs within Kids First and Odyssey House Victoria and external services such as the statutory child protection service, and other Melbourne-based DV and AOD services.

Fathers entering KODY or ‘*Caring Dads* standard’ programs will be assessed and invited at intake by program staff to participate in the evaluation, as will men’s (ex)partners. Researchers will follow up with interested participants to obtain informed consent which will be confirmed at each stage of data collection. Program staff will be consulted regarding any possible safety issues.

Three KODY groups and three ‘*Caring Dads* standard’ groups are planned for each year of the study, each with up to 10 participants. Assuming a medium effect size (based on Cohen’s d), a power of 0.80 and a two-tailed test across independent groups, this study will need to involve reports from 128 mothers, 64 in each of the KODY and comparison groups. Reported participation rates for evaluations of *Caring Dads* programs range from 62% [3] to 97% [5]. Assuming a partner contact success rate of 75% based on the benefits of the *Kids in Focus* program for women and children, this study will need to enrol 170 families across groups.

### Primary outcomes and measures

The primary outcome is reported improvements in the safety and wellbeing of women and children affected by the behaviour of KODY participants. Primary and secondary outcomes, and proposed measures, are outlined in Table 1. The measures will be tested for feasibility during the program pilot and then implemented with necessary adjustments during the trial proper. Measures have been selected in consultation with program staff to ensure their suitability for program outcomes. Where appropriate, brief versions of measures were selected to reduce participant burden, as well as measures that were already used for assessment by program staff. A range of validated and unvalidated measures were selected as validated measures identified in the literature alone were not seen to adequately measure outcomes.

#### Composite abuse scale

The primary outcome of improved safety and wellbeing of women is measured by the Composite Abuse Scale Standard Form [33], a 30-item self-report measure completed by the mothers involved in the KODY evaluation to identify the nature and severity of their (ex)partner’s abusive behaviour. Statements describing partner behaviours are scored on a 6-point scale (‘never’ to ‘daily’) to assess the frequency of these behaviours across time. Statements reflect four categories of abuse: severe combined abuse, emotional abuse, physical abuse and harassment. A reduction in scores represents a decrease in women’s experiences of these (ex)partner behaviours.

Several measures are used to assess the primary outcome of improved safety and wellbeing of children. These include the Emotional Dysregulation Scale, the Interpersonal Mindfulness in Parenting scale, the Brief Infant–Toddler Social and Emotional Assessment and the Strengths and Difficulties Questionnaire, as outlined below.

#### Emotional dysregulation scale

The Emotional Dysregulation Scale [34] is a 12-item validated measure used to determine overall individual emotional dysregulation. This scale was selected considering research showing that children are less safe when their fathers’ emotions are dysregulated [35], particularly in the context of substance use [36], as well as in recognition of *Caring Dads*’ focus on explicitly teaching skills related to emotion regulation. In the KODY evaluation, fathers answer questions about their own emotion regulation abilities, while mothers are asked to report on their (ex)partner’s emotion regulation abilities. Items are scored on a 7-point scale (‘not true’ to ‘very true’). Items assess domains of emotional experiencing, cognition and behaviour.

#### Interpersonal mindfulness in parenting scale

The Interpersonal Mindfulness in Parenting Scale [37] is a 27-item self-report scale that measures mindfulness in the parenting context. This scale aligns with ‘child-centred fathering’, a concept central to the *Caring Dads* program, with items suitable for assessing fathers’ skill improvement, as well as their ability to regulate their emotions when parenting, complementing findings from the Emotional Dysregulation Scale. Items are scored on a 5-point scale (‘never true’ to ‘always true’). Items assess mindful parenting across five dimensions: listening with full attention, non-judgmental acceptance of self and child, compassion for self, emotional awareness of self and child, and self-regulation in parenting.

#### Strengths and difficulties questionnaire

The Strengths and Difficulties Questionnaire [38] is a 25-item validated measure that assesses child mental health in children aged 3–16 years. In the KODY evaluation, this questionnaire will be completed by mothers to measure improvements in children’s mental health and wellbeing over time. Mothers use a 3-point scale (‘not true’ to ‘certainly true’) to indicate how well a range of statements describe their child. These statements describe child behaviours across five domains: emotional problems, conduct problems, hyperactivity, peer relationship problems, and prosocial behaviour. This scale was selected for the KODY evaluation to complement the Brief Infant–Toddler Social and Emotional Assessment by assessing child wellbeing across an older age group. Adolescents involved with KODY will also be invited to complete this measure.

#### Brief infant–toddler social and emotional assessment

The Brief Infant–Toddler Social and Emotional Assessment [39] is a 42-item parent-report validated measure used to screen for social-emotional and behavioural problems and developmental delay in children aged 1–3 years. In the KODY evaluation, mothers will use a 3-point scale (‘not true/rarely’ to ‘very true/often’) to indicate how well a range of statements describe their child’s behaviour. These statements describe child behaviours across two subscales: the problem scale, measuring behaviours that, if present, represent a problem; and the competence scale, measuring behaviours that, if absent, represent a problem. This scale complements the Strengths and Difficulties Questionnaire by assessing child wellbeing in a younger age group.

### Secondary outcomes and measures

The secondary outcome of fathers’ use of DV relates directly to the primary outcomes of improved safety and wellbeing for women and children, as DV is seen as a major risk. Further, the secondary outcome of fathers’ use of AOD, will also contribute to the primary outcomes, given the evidence that where fathers use both DV and AOD, the severity of abuse, and therefore the risk to safety is significantly higher [1]. The secondary outcome of fathers’ use of DV will be assessed through reporting from fathers and mothers, using an adapted Mirabal measure, the Anger Management Scale, and the Brief Irritability Test.

#### Adapted mirabal measure

For the KODY evaluation, the research team has created an 18-item measure, adapted from an existing measure used in Project Mirabal, a multi-site longitudinal study of domestic violence perpetrator programs, conducted between 2009 and 2015 in the UK [40]. The adapted measure has a version for mothers and one for fathers, in order to assess and compare mothers’ and fathers’ perceptions of fathers’ behaviour change over time. Mothers and fathers respond to a range of statements on a 4-point scale (‘strongly disagree’ to ‘strongly agree’).

The adapted Mirabal uses items from four of the six indicators of change outlined in Project Mirabal, asking mothers to assess their (ex)partner’s respectful communication, fathering, awareness of self and others, and their own perceptions of their children’s experiences (e.g. ‘My children worry about the safety of their mother’). Fathers who have used violence are provided with a similar range of statements, across the same domains.

#### Anger management scale

The Anger Management scale is a 12-item subscale of the Personal Relationships Profile, a validated measure developed by Straus and colleagues [41]. This scale asks fathers to assess their ability to recognise and control their anger towards the mother of their children. It was used with fathers in the Victorian *Caring Dads* trial [3], and found an overall increase in men’s scores from baseline to post-program time points, indicating an improvement in men’s ability to recognise and control their anger after completing the *Caring Dads* program. Items are scored on a 4-point scale (‘strongly disagree’ to ‘strongly agree’) and includes items from three subscales: behavioural self-soothing, recognising signs of anger and self-talk.

#### Brief irritability test

The Brief Irritability Test [42] is a 5-item scale that measures the degree to which respondents experience frustration and irritability. This scale was selected for use with fathers in the KODY evaluation to complement the Anger Management scale. Irritability is similar to anger, but often persists for longer and is outwardly expressed in the form of aggressive behaviour [42]. As high levels of irritability are also associated with higher levels of stress, this measure was selected to provide some insight into fathers’ wellbeing. Items are scored on a 6-point scale (‘never’ to ‘always’) to indicate how frequently respondents identify with each statement, considering their feelings over the past two weeks. The secondary outcome of fathers’ use of AOD will be assessed through reporting from fathers, using the Alcohol Use Disorders Identification Test and the Drug Use Disorders Identification Test.

### Alcohol use disorders identification test and drug use disorders identification test

The Alcohol Use Disorders Identification Test (AUDIT) [43] is a 10-item self-report screening tool developed by the World Health Organization to assess alcohol consumption, drinking behaviours and alcohol-related problems. The Drug Use Disorders Identification Test (DUDIT) [44] is an 11-item self-report screening tool, developed as a parallel instrument to the AUDIT, to assess use of drugs other than alcohol and drug-related problems. Both the AUDIT and the DUDIT have been validated across genders and in a wide range of culturally and linguistically diverse groups. These tools were selected to provide insight into fathers’ AOD use.

### Data collection

The outcomes evaluation will involve data collection at four timepoints (see Table 2). Psychometric tests will be administered to men and women participants at three timepoints—baseline (T1), post-*Caring Dads* program (T2) and three months after the end of the *Caring Dads* program (T3) (see Table 2). Men and women will receive a small honorarium at each point of data collection to recognise the donation of their time.

**Table 2:** Data Collection Timeline

Data Collection		Fathers	Mothers
Baseline	T1	▪Measures	▪Measures
post-*Caring Dads* program	T2	▪Measures ▪File Review ▪Process evaluation	▪Measures ▪File Review ▪Process evaluation
3-month follow up	T3	▪Measures ▪File Review ▪Process evaluation	▪Measures ▪File Review ▪Process evaluation
9-month follow up	T4	▪File Review to identify subsequent abuse concerns	▪File Review to identify subsequent abuse concerns

File reviews of KODY participants will be undertaken at T2, T3 and at 9-month follow up (T4) to obtain demographic data about participants, as well as program data, such as ‘dose’ level of expanded service, referral sources, other services involved with clients and accountability arrangements (e.g. court orders).

### Data management

Program participants and professionals will be assigned a unique research ID to ensure anonymity. Outcome measures will be completed online and stored in a secure Qualtrics database. Other research data will be stored in the University of Melbourne OneDrive system with access restricted to members of the research team. Interviews and focus groups will be audio-recorded with participant consent. If these are conducted online, recording will be through Zoom and saved directly into OneDrive. Recordings of face-to-face interviews and focus groups will also be uploaded onto OneDrive. Password protection on files will be used as required.

### Data analysis

Intent to treat analyses will be used so that all service users are included in the primary analysis, allowing for variations in levels of participation in KODY and including those who drop out prematurely. Assuming differences between groups, supplementary analyses will examine the extent to which intervention effects vary according to whether families received a higher or lower ‘dose’ of expanded service. Recognizing that fathers are not being randomly assigned to the intervention and comparison group, care will be taken to compare time 1 characteristics of groups and, if necessary, use propensity analyses in post-group and follow-up comparisons. Each component of the study will be analysed separately in the first instance, then brought together to triangulate data collection and analysis, before a final synthesis.

## Discussion

This paper outlines the protocol for a quasi-experimental trial of the innovative KODY program, an all-of-family response to co-occurring issues of DV and AOD. A quasi-experimental design was deemed most appropriate for the trial, as randomized allocation to trial and comparison groups is impracticable and potentially unethical in the context of the program and the service system in which it operates. Primary outcomes for the study are the increased safety and wellbeing of mothers and children whose (ex)partners and fathers respectively participate in KODY (the trial program), when compared with ‘*Caring Dads* standard’ (the comparison group).

Through an analysis of both outcomes measures and file reviews to provide ‘dose’ level information, the study aims to build the evidence for program responses that increase the safety and wellbeing of women and children impacted by men who use DV and AOD. The innovative aspects of the program—addressing the co-occurrence of DV and AOD with a specific focus on fathering, providing support to all members of the family and centring the needs of children—are all drawn from both academic evidence and practitioner expertise. The innovative nature of the program may result in small sample sizes in the early years of implementation, which may limit the power of analyses, but the authors’ experience with the evaluation of other innovative programs suggests that sample sizes will increase with program longevity. Operating within the DV and AOD service sectors respectively, the two partner organisations—Kids First and Odyssey House Victoria—have a shared focus on the needs of vulnerable children and train their staff in a common framework (Safe and Together) which brings a shared language and conceptualisation of the intersection of AOD and DV [30]. In the study context of Victoria, Australia, their joint commitment to collaborating across these sectors exemplifies a shift in the service system context and policy drivers away from a siloed service system which results in separate and disconnected service responses, particularly when children are involved [21].

## Data Availability

The datasets used and/or analysed during the current study will be available from the corresponding author upon reasonable request.

## Abbreviations

KODY: The name of the program is an acronym based on the names of the collaborating Organisations: Kids First (Caring Dads) and Odyssey House Victoria.
DV: Domestic violence
AOD: Alcohol and other drugs
MBC programs: Men’s behaviour change programs
T1, T2, T3, T4: Time 1, Time 2, Time 3, Time 4
AUDIT: Alcohol Use Disorders Identification Test
DUDIT: Drug Use Disorders Identification Test
